# Positive Predictive Value for Multitarget Stool DNA After Bariatric and Metabolic Surgery

**DOI:** 10.1016/j.gastha.2023.06.005

**Published:** 2023-07-01

**Authors:** Derek W. Ebner, Kelli N. Burger, Brendan Broderick, Douglas W. Mahoney, Todd A. Kellogg, Andres Acosta, John B. Kisiel

**Affiliations:** 1 Division of Gastroenterology and Hepatology, Mayo Clinic, Rochester, Minnesota;; 2 Division of Clinical Trials and Biostatistics, Mayo Clinic, Rochester, Minnesota;; 3 Division of Endocrine & Metabolic Surgery, Mayo Clinic, Rochester, USA Minnesota

**Keywords:** Bariatric and Metabolic Surgery, Colorectal Neo-plasms/Prevention and Control, DNA Methylation, Colonoscopy

## Abstract

**BACKGROUND AND AIMS:**

Bariatric and metabolic surgery (BMS) may adversely affect noninvasive stool tests for colorectal cancer (CRC) screening through several mechanisms. Multitarget stool DNA (mt-sDNA) is approved for CRC screening; however, performance in post-BMS patients is unknown. As the rates of BMS are anticipated to increase with rising incidence of obesity, it is important to evaluate mt-sDNA test performance among these patients.

**METHODS:**

In a multisite academic and community-based practice, we obtained mt-sDNA results from 10/2014 to 12/2019 through electronic records and an institutional BMS registry. Average CRC risk patients with BMS prior to a positive mt-sDNA underwent a detailed chart review. Follow-up colonoscopy findings were compared to those among BMS patients screened with colonoscopy alone and a historical cohort of patients without BMS, screened by mt-sDNA. The primary study endpoint was the positive predictive value (PPV) for advanced colorectal neoplasia.

**RESULTS:**

Among 336 average-risk patients who had mt-sDNA after BMS, mt-sDNA was positive in 49 (14.6%), 47/49 (96%) underwent follow-up colonoscopy, and the PPV for advanced neoplasia was 12/47 (25.5%). This is similar to the PPV for advanced colorectal neoplasia (425/1542, 28%) in a historical cohort of persons without prior BMS, screened by mt-sDNA at our center (*P* = .86). Among those who had prior BMS, the rate of advanced neoplasia was higher after mt-sDNA compared to screening colonoscopy alone.

**CONCLUSION:**

Despite anatomic and physiologic mechanisms that could alter blood or DNA content in stool, BMS does not appear to adversely affect the PPV of mt-sDNA.

## Graphical Abstract

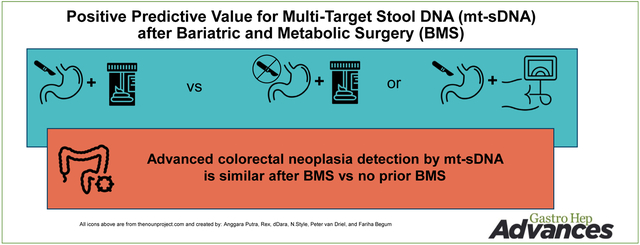

## Introduction

The prevalence of obesity is rising and presently exceeds over 40% in the United States.^1^ For people with obesity, there is increased morbidity/mortality,^2^ healthcare utilization, sick days, and work productivity losses.^3–5^ Despite public service initiatives,^6^ the prevalence of people with obesity is projected to reach 50% by 2030.^7^ To manage the obesity epidemic, a multidisciplinary approach may include bariatric and metabolic surgery (BMS).^8^ BMS has demonstrated efficacy in reversing obesity complications, including diabetes, hypertension, and sleep apnea.^9^ Furthermore, BMS has prospectively demonstrated improved overall survival.^10^ Accordingly, the utilization of BMS has been increasing^11,12^ and will likely continue to rise.^13^

Weight-based disparities in the delivery of preventative health services^14^ and preventative health care avoidance^15^ have been observed among adults with obesity.^16,17^ Cancer screening is particularly important as obesity confers an increased risk for different cancer types including colorectal cancer (CRC).^18^ Over a third of age-eligible obese adults remain unscreened.^19^ Reasons for this are manifold and may include incomplete screening colonoscopy.^20^ Noninvasive CRC screening may be particularly helpful among adults with obesity and has been shown to increase adherence to CRC screening in a general population.^21^ Among the United States Multi-Society Task Force endorsed screening modalities, a recent patient survey demonstrated a preference for multitarget stool DNA (mt-sDNA).^22^ mt-sDNA detects methylated *BMP3* and *NDRG4*, mutant *KRAS*, *β-actin*, and hemoglobin; a locked multiparameter algorithm is used to report a positive or negative test result.^23^

**Figure 1.**
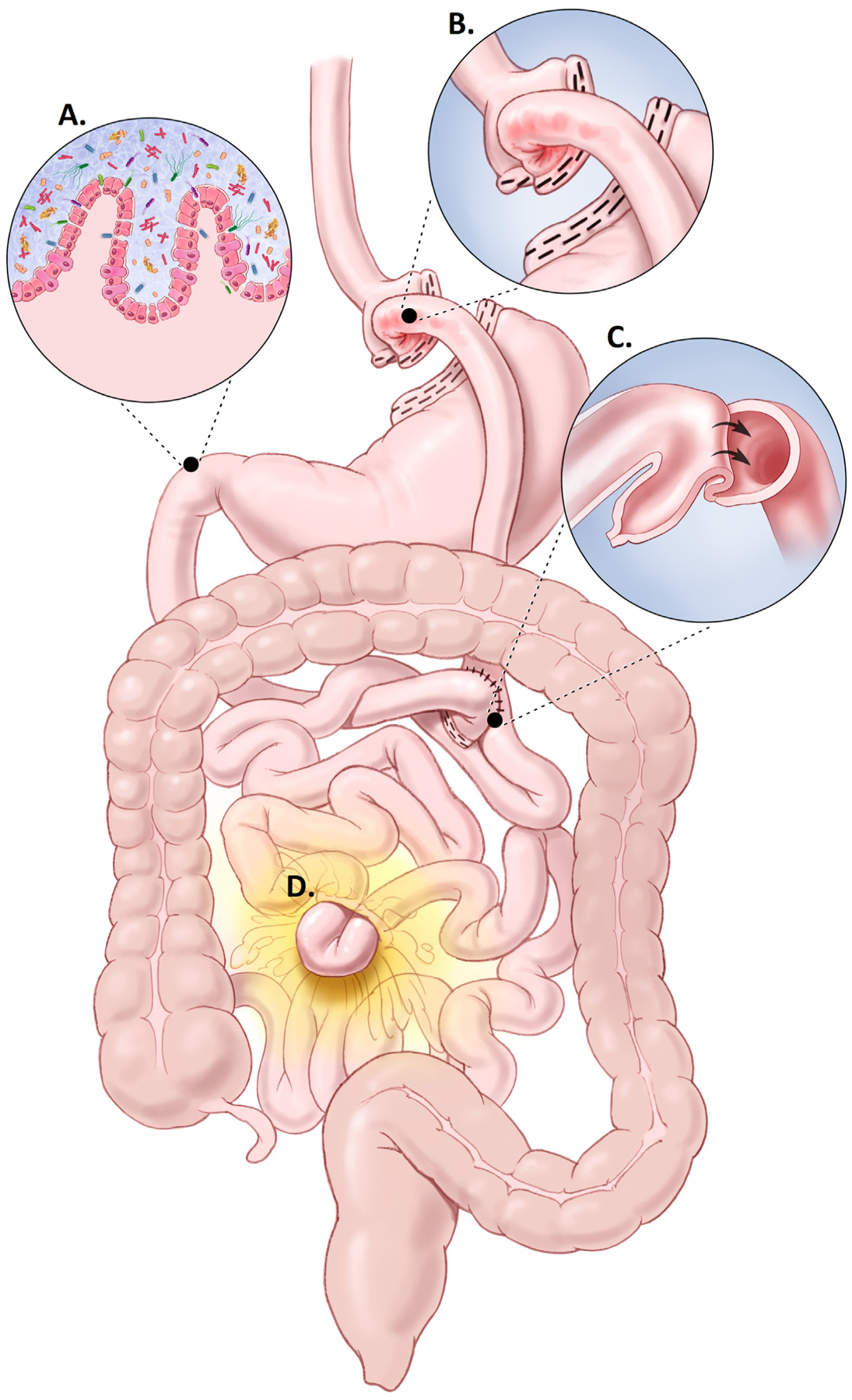
Roux-en-Y characteristics that may alter mt-sDNA test results: (A) Voluminous stool output related to bacterial overgrowth, altered gut microbiome, or dumping; (B) Mucosal disruption/bleeding from anastomotic ulceration; (C) Intussusception; or (D) Internal herniation. Used with permission of Mayo Foundation for Medical Education and Research, all rights reserved.

Enthusiasm for noninvasive CRC screening may be dampened by consideration of anatomic and physiologic consequences of BMS could theoretically alter stool blood or DNA content (Figure 1). Marginal ulcers are common with an incidence ranging from 0.6% to 25%.^24^ Gastrointestinal bleeding from marginal ulcers or other post-BMS complications like internal hernias^25^ and intussusception^26^ could be detected by a stool-based screen, lead to a false positive, and unnecessary use of follow-up colonoscopy. Among those with BMS, iron deficiency is also common^27,28^ and classically warrants diagnostic colonoscopy to exclude CRC as the source. Additionally, post-BMS diarrhea from dumping, bile acid malabsorption, or bacterial overgrowth could uniquely affect mt-sDNA. The mt-sDNA collection kit provides a buffer solution that stabilizes the stool sample for molecular processing.^23,29^ Voluminous stool may exceed the buffering capacity of the solution and altered bowel transit may influence exfoliation of tumor markers, altering test performance.

Although patients with prior BMS have not been excluded from key studies evaluating mt-sDNA,^30–32^ the impact of BMS on this test has not been directly studied. With the increased utilization of BMS,^33^ it is important to evaluate whether BMS adversely affects mt-sDNA performance as this could lead to costly overutilization of colonoscopy. We therefore aimed to describe factors leading to mt-sDNA selection among those with BMS and report findings at follow-up colonoscopy to estimate positive predictive value (PPV). The PPV was then compared to a historical cohort of patients from the same institution^34^ who were at average risk for CRC, utilized mt-sDNA, and had no history of BMS. While this comparison provides a direct way to evaluate whether BMS changes mt-sDNA test performance, in the real world those with a negative mt-sDNA do not undergo colonoscopy. To account for rates of neoplasia among those with BMS, the PPV among those with BMS and subsequent utilization of colonoscopy alone (no use of mt-sDNA) was also compared.

## Methods

After institutional review board approval, we reviewed records to identify patients with prior BMS who had an mt-sDNA test between 10/2014 and 12/2019 at any of the Mayo Clinic sites (Jacksonville Florida, Phoenix Arizona, Rochester Minnesota, and the tri-state Mayo Clinic Health System (Southern Minnesota, Western Wisconsin, and Northern Iowa)). In accordance with Minnesota law, patients who had not consented to research (including chart review) were excluded.

### Population and patients

We identified patients who had BMS using 2 search strategies. The first queried a database maintained by the Division of Bariatric Surgery of their operations since May 1, 2008 (Table A1). Second, we performed a search of the shared electronic medical record (EMR, Epic Systems [Verona, WI]) using codes from the 10th revision of the International Statistical Classification of Diseases and Related Health Problems and structured free-text search terms of past medical history/diagnosis of all available records. The search terms were applied to a subset of the data and the terms were ranked, based on the number of patients correctly identified in a manual chart review, before inclusion in the final search algorithm. Patient lists were merged, and unique patients identified.

Then, the Mayo Clinic central data warehouse was utilized to search mt-sDNA lab/order, procedure, and diagnosis codes to identify patients with an mt-sDNA test result. The collective list of patients with a suspected history of BMS was cross-referenced to mt-sDNA testing over the study period. Patients without mt-sDNA use or mt-sDNA testing dated before a known surgical date for BMS were excluded. The remaining cohort underwent a chart review.

### Study endpoints and data collection

The primary study endpoint was the overall test positivity rate and the PPV for mt-sDNA among post-BMS patients at average CRC risk (*mt-sDNA after BMS* cohort) in comparison to BMS patients with screening colonoscopy only (*screening colonoscopy after BMS* cohort) and historical data from persons screened by mt-sDNA and no history of BMS (*non-BMS mt-sDNA* cohort). Secondary analyses included stratification of the primary endpoints for presence of anemia, history of marginal ulcers, and current nonsteroidal anti-inflammatory drug (NSAID) use. Where possible, we abstracted any record of patient/provider decision-making for mt-sDNA use.

Review of each patient’s EMR was done by one reviewer (D.W.E.) and the data were recorded and stored using REDCap (Vanderbilt University, Nashville, TN). For the *mt-sDNA after BMS* cohort, patients with mt-sDNA use before BMS, no history of BMS, or minimally invasive BMS (endoscopic or gastric band) were excluded (Table A1).

Baseline characteristics included patient demographics, tobacco use, and past medical and family history to assess average vs increased risk for CRC.^30^ Anemia within 3 months of mt-sDNA was also recorded and classified (iron deficiency, B12/folate deficiency, chronic disease, or other). Given the high prevalence of iron deficiency among those with BMS those with iron deficiency were included in the composite analysis and subsequently evaluated as a subgroup.

CRC screening by colonoscopy prior to mt-sDNA use and reports of any history of neoplasia were evaluated (hyper-plastic polyp, tubular adenoma, sessile serrated lesion, degree of dysplasia, CRC) and the highest-graded lesion was recorded. The date and type of BMS were recorded including presurgical body mass index (BMI), history of revision, marginal ulcerations, NSAID use, or features of malabsorption (dumping syndrome, diarrhea, use of antimotility agents, pancreatic enzymes, or bile acid binders) within 3 months prior to mt-sDNA.

The date of mt-sDNA and result (positive or negative) were noted. In review of encounter notes, the indication for mt-sDNA use was determined based on available notation. Broadly, these were categorized as: 1) shared decision-making, if specific notation was available reviewing the various options for CRC screening; 2) patient choice, if there was notation that the patient specifically requested the mt-sDNA test in the absence of reviewing alternative methods; 3) provider preference, given a concern for tolerance of colon preparation or opinion the patient was not suitable for colonoscopy; or 4) unknown.

The findings at follow-up colonoscopy were also enumerated (polyp architecture, size, number, dysplasia severity, and colonoscopy preparation quality). Hyperplastic polyps, irrespective of size, were excluded as neoplasia when determining PPV. Test PPV for neoplasia (CRC, advanced neoplasia, and non-advanced neoplasia) was then compared to 2 independent patient cohorts (*screening colonoscopy after BMS* and *non-BMS mt-sDNA*).

### Comparative cohorts

The first control group included a previously published cohort at our institution, which included average-risk adults, screened by mt-sDNA from October 2014 through December 2017.^34^ Those with a history of BMS were included only in the *mt-sDNA after the BMS* group.

A second comparison was to patients with BMS history who underwent screening colonoscopy during the study period. To generate the *screening colonoscopy after the BMS* comparative group, the list of patients with prior BMS who did not have an mt-sDNA test result were queried in the institutional endoscopy database (ProVation Minneapolis, Minnesota). Those with a colonoscopy indication of average risk screening during the study period (10/2014 through 12/2019) were included. The colonoscopy indication at our institution is entered by the performing endoscopist and accuracy has been internally validated using manual chart review.^35^ Therefore, manual chart review did not revalidate patient risk or evaluate prior screening history. All available patients were manually reviewed to verify BMS history, absence of mt-sDNA results, and detailed results of colonoscopy screening.

### Analysis plan

Patient characteristics among the cohorts *mt-sDNA after BMS*, *screening colonoscopy after BMS*, and *non-BMS mt-sDNA* were compared using the Wilcoxon rank sum test for continuous variables (summarized as a median with corresponding 25th and 75th percentiles) whereas proportions were compared by the Fisher’s exact test. PPV was calculated as the number of positive findings (excluding hyperplastic polyps) relative to the total number of patients undergoing follow-up colonoscopy with corresponding 95% confidence intervals. The required sample size to detect an odds ratio of 2 (absolute difference of ±15%) between 2 independent cohorts with 80% power was estimated to be 150 patients per group assuming a reference PPV of 60% and a two-sided significance level of 0.05. However, the actual number of BMS patients with a positive (+) *mt-sDNA* test who underwent colonoscopy was less than anticipated. Given the observed sample sizes, the minimum detectable odds ratio with the same level of power, significance, and reference PPV increased to 2.6 (absolute difference of ±20%).

## Results

### Patient characteristics: mt-sDNA after BMS

**Figure 2.**
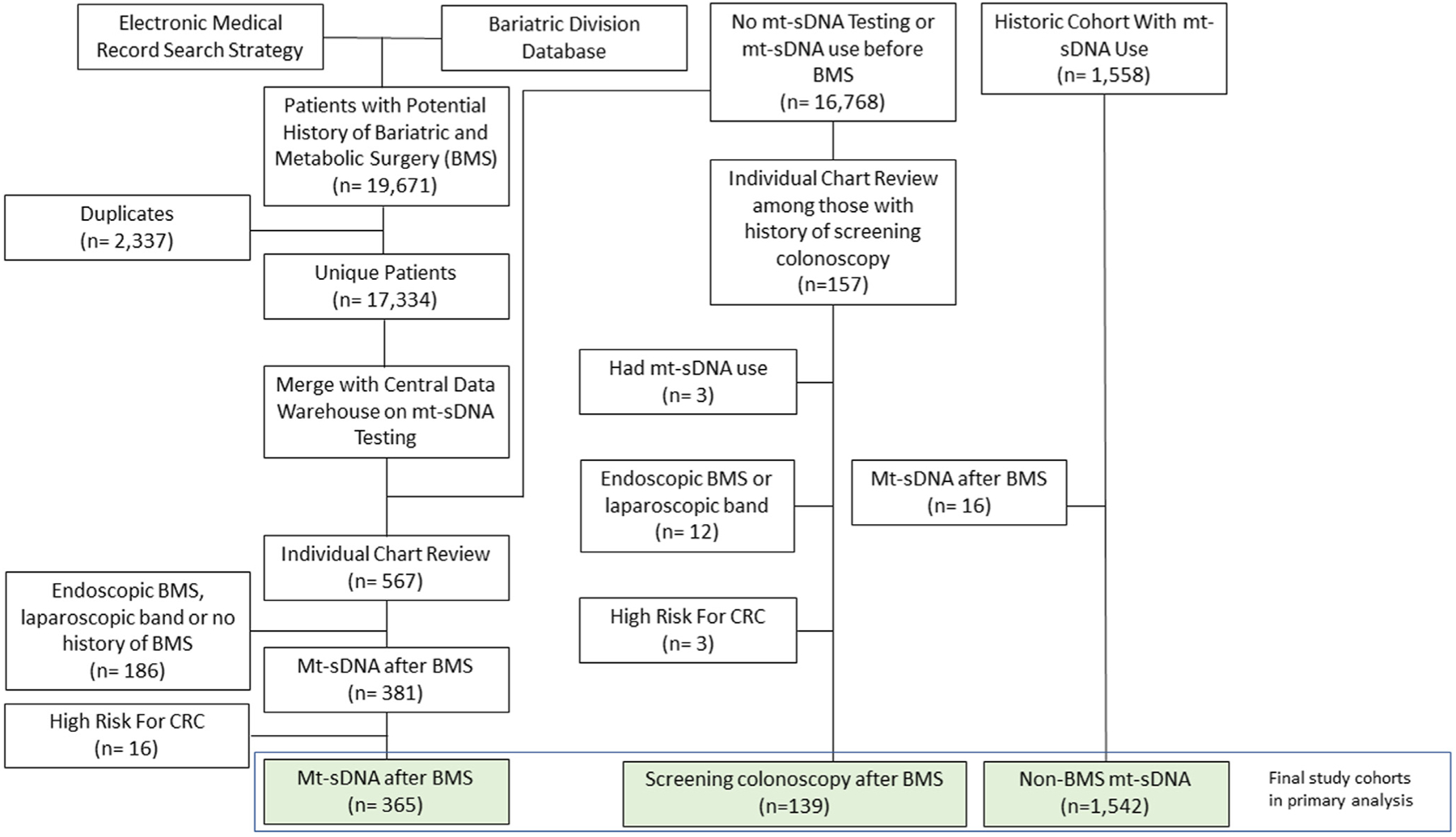
Study flow diagram for bariatric and metabolic surgery (BMS) cohort generation among those utilizing multitarget stool DNA (mt-sDNA) vs colonoscopy for colorectal cancer (CRC) screening.

The EMR search heuristic and divisional database combined included 17,334 unique patients with a potential history of BMS. After excluding those who had never undergone mt-sDNA or who had mt-sDNA use before BMS, 567 patients remained and underwent dedicated chart review. There were 381 patients confirmed to have undergone BMS with subsequent mt-sDNA, of which there were 16 patients who had high-risk features for CRC (specifically, family history of CRC in a 1st degree relative <60 years of age, personal history of high-risk colorectal neoplasia, aero-digestive cancer within 5 years of mt-sDNA, rectal bleeding or positive fecal blood testing within previous 6 months of mt-sDNA). The final cohort (*mt-sDNA after BMS*) consisted of 365 average-risk patients, with or without iron deficiency 90 days prior to mt-sDNA use (Figure 2).

Of the 365 patients, most were women (84%), white (99%), and 209 (57%) had a history of colonoscopy prior to mt-sDNA. Among 14 with findings at prior colonoscopy; most were hyperplastic lesions and only 3 patients had precancerous polyps, all of which were low-risk.^36^ The median BMI prior to BMS was 44 (40–49) kg/m^2^ and the median time from BMS to mt-sDNA was 8.9 (4.9–14.7) years. Roux-en-Y gastric bypass was conducted for 297/365 (81%). A history of marginal ulcers was seen 27/365 (7%), and 22/361 (6%) had prior surgical revision. Additional patient characteristics are noted (Table 1).

### Patient characteristics: comparative cohorts

There were some differences in patient characteristics (Table 1). Those screened with mt-sDNA were older. There were more women in the BMS groups. Among those screened with mt-sDNA, those without BMS had a higher percentage of prior colorectal neoplasia.

### PPV of mt-sDNA after BMS without iron deficiency anemia

The mt-sDNA positivity rate for the *mt-sDNA after BMS* cohort, independent of CRC risk, was 59/381 (15.5%). Among the final study cohort of 365 otherwise average risk post-BMS patients, 29 had iron deficiency anemia. Of the 336 average-risk patients without iron deficiency anemia, mt-sDNA was positive in 49 (14.6%), 47/49 (96%) underwent follow-up colonoscopy, and most had a Roux-en-Y (41/47, 87%). For the 47 patients who underwent follow-up colonoscopy bowel preparation was not reported for one and was otherwise adequate for all but 5/46 (11%). Cecal intubation was achieved for 46/47 (98%). At least one neoplasm was found in 29 of the 47 patients who had a follow-up colonoscopy (PPV 61.7%, 95% CI [46,75]). Of total neoplasia detected, most included findings in the right colon (23/29, 79%). The PPV for advanced neoplasia was 12/47 (25.5%, 95% CI [14,40]).

### Comparative findings

**Figure 3.**
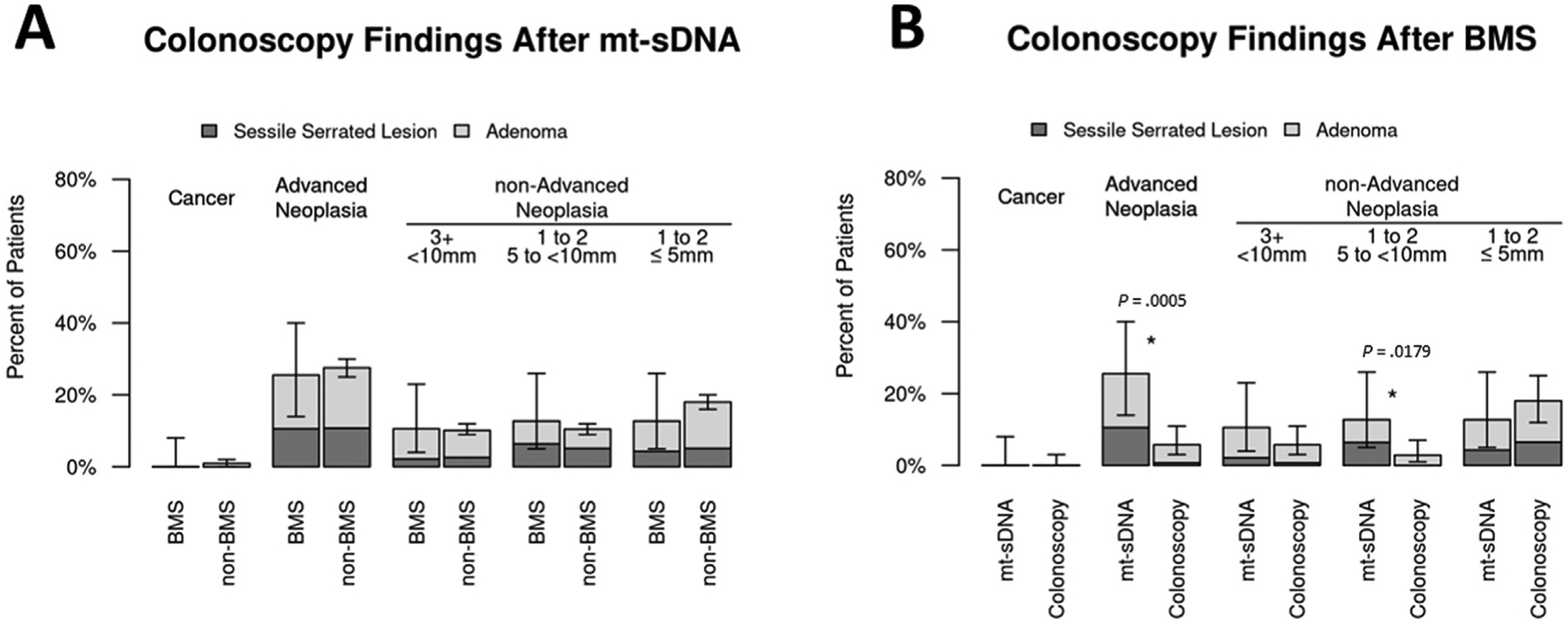
Proportion of neoplasia found at positive (+) multitarget stool DNA (mt-sDNA) colonoscopy among average-risk patients with bariatric and metabolic surgery (BMS) compared to: (A) average-risk patients with +mt-sDNA and no history of BMS, (B) BMS patients with screening colonoscopy alone.

When those with *mt-sDNA after BMS* were compared to the *non-BMS mt-sDNA* cohort, there were no differences in the diagnosis of CRC, advanced, or non-advanced neoplasia (Figure 3A, Table A2). However, differences were observed when the *mt-sDNA after BMS* and *screening colonoscopy after BMS* cohorts were compared (Figure 3B). The rate of advanced neoplasia was significantly higher after a +mt-sDNA in comparison to patients who underwent screening colonoscopy alone. Among the BMS cohorts, colonoscopy preparation quality was no different although the colonoscopy withdrawal time in those without neoplasia after a +mt-sDNA was significantly longer (Table A3).

### PPV with iron deficiency, history of marginal ulceration, and NSAIDs

Among BMS patients who were otherwise at average risk for CRC, anemia was present for 41/365 (11%). The majority of these were secondary to iron deficiency 29/41 (71%). Among those with iron deficiency, 5 (17%) had a +mt-sDNA; 4/5 underwent follow-up colonoscopy; and 1/4 had non-advanced neoplasia only. Of the 29 patients with iron deficiency, 5 had a history of marginal ulceration and mt-sDNA testing was negative for each of those patients. For the 27/365 patients with a history of marginal ulcers, mt-sDNA was positive for 3/27 (11%); follow-up colonoscopy detected an advanced adenoma for one patient and colonoscopy was normal for the other 2 patients.

Of the additional potential confounders, malabsorption was only suggested for 4% (15/365) of the cohort; additionally, NSAID use within 3 months of mt-sDNA was observed in 32% (115/365). Among the 115 NSAID users, 10 (9%) had iron deficiency anemia and 23 (20%) had a +mt-sDNA. Follow-up colonoscopy was pursued for 20/23 (87%) and neoplasia was diagnosed for 12/20 (60%). Among those with neoplasia, advanced neoplasia was diagnosed for 5/12 (42%). An additional patient had 2 polyps removed but the tissue was not retrieved and was therefore not counted as neoplasia.

### Decision-making

A process of shared decision-making about the modalities for CRC screening was documented prior to mt-sDNA screening in 315/381 (82.6%). Specific notation of patient or provider concerns about bowel preparation tolerance was recorded for 17/381 (4%) of those with BMS who were screened by mt-sDNA.

## Discussion

There is theoretical concern regarding the use of mt-sDNA among those with a history of BMS given the high prevalence of iron deficiency, risk for marginal ulceration with subsequent gastrointestinal hemorrhage, and increased risk for CRC. We observed that the test positivity (14.6%) and PPV of 25.5% and 61.7% for advanced precursors and any colorectal neoplasia, respectively, are highly consistent with values observed in patients without BMS. The test positivity of mt-sDNA has ranged from 14% to 16% in real-world observational study among patients with mixed risk for CRC^34^ and prospective evaluation of average-risk individuals.^30^ Thus, mt-sDNA does not appear to generate a meaningful increase in false positive mt-sDNA tests in patients with prior BMS, who are otherwise at average risk for CRC.

The rate of mt-sDNA use after prior colonoscopy among those with prior BMS appears to mirror non-BMS patients at our institution; the mt-sDNA after BMS cohort rate of prior colonoscopy was 57% compared to 59% at our institution for average-risk adults.^34^ However, there was significantly higher neoplasia at prior screening colonoscopy among the non-BMS group compared to those with BMS, and the *non-BMS mt-sDNA* screened patients were significantly older. Despite these differences in patient characteristics, mt-sDNA test performance was similar. Of note, in the BMS cohort, there were 156 average-risk patients without a history of colonoscopy prior to mt-sDNA use and 122 were overdue to start CRC screening. We observed that the rate of attendance for follow-up colonoscopy after a +mt-sDNA for those with BMS history (51/54, 94%) is similar to published reports that range 85%–96%.^34,37,38^ Among the BMS cohort, mt-sDNA detected high rates of right-sided and sessile serrated neoplasia, similar to previous reports.^31,34,39,40^ In the pivotal prospective study of mt-sDNA with criterion colonoscopy among average-risk individuals, the test positivity rate was 16% and advanced neoplasia PPV was 23.6%^30^; in the present study, the test positivity among average-risk BMS was 14.6% and advanced neoplasia PPV was 25.5%. When patients with BMS and iron deficiency anemia are included, test positivity is essentially the same (54/365, 14.8%). We reinforce that mt-sDNA should not be used and is not approved as a test for evaluating the underlying cause of iron deficiency anemia.

While our evaluation comes from the detailed chart review, this was a retrospective cohort study, which can introduce biases and limitations. As patients with a negative mt-sDNA do not undergo colonoscopy in real-world conditions, our primary analysis focused on test positivity and the PPV; negative predictive value could not be calculated. To address this limitation, we compared neoplasia rates among post-BMS patients undergoing colonoscopy alone. In an effort to account for colonoscopy performance that can otherwise change overtime, the BMS cohorts (mt-sDNA and colonoscopy alone) were evaluated over the same study period. Our retrospective study design limits our ability to elaborate on the lower rate of advanced neoplasia among the screening colonoscopy after BMS group when compared to mt-sDNA after BMS. However, this observation may be related to increased post-test probability after a positive mt-sDNA, variability in prior CRC screening between the groups or other patient-level factors. For example, we did not have control over which patients utilized mt-sDNA and were unable to measure BMI at the time of screening. We were able to determine that shared decision-making between patients and providers was a key driver in mt-sDNA use, but we cannot account for all reasons that influenced use. The manner in which BMI at the time of CRC screening influences patient decision-making on invasive vs noninvasive testing warrants further study. These data could help inform strategies for CRC screening across a community and help tailor dialogue surrounding screening strategies to optimize utilization. Our findings are most applicable to patients with a history of Roux-en-Y gastric bypass (Table 1); subgroup analysis of non-Roux-en-Y patients was under powered. Demographic differences between mt-sDNA users with and without prior BMS are noted. For example, BMS patients were predominantly female and white; however, these findings represent the anticipated demographic for individuals undergoing BMS in the United States.^41,42^ Additionally, more women in the BMS group and older patients in the non-BMS group would both bias toward a *lower* rate of colorectal neoplasia diagnosis in those having BMS before mt-sDNA, which was not observed. This may suggest that the history of obesity or BMS may independently raise the prevalence of colorectal neoplasia. However, this observation may also reflect the low diagnosis rate of neoplasia at historic colonoscopy prior to the use of mt-sDNA, which is beyond the aim of study to evaluate further. Despite an extensive search heuristic rendering the largest cohort of patients with BMS undergoing mt-sDNA, we acknowledge the sample size of test-positive patients is small. Lastly, our study population was mostly white; this potential barrier to generalizability is mitigated, at least in part, by multiple prior studies which show that race does not appear to influence the markers and results of the mt-sDNA test.^29–31,43,44^

BMS is a key strategy in the management of obesity. While there are theoretical concerns that post-BMS anatomy and physiology may alter the performance of mt-sDNA, contribute to false-positive tests, and overutilization of follow-up colonoscopy; these concerns were not supported. The performance of mt-sDNA is similar to that observed among average-risk adults and can be reliably utilized for CRC screening.

## Supplementary Material

1

2

3

## Figures and Tables

**Table 1.** Patient Characteristics Among Average-Risk Adults With Bariatric and Metabolic Surgery (BMS) Followed by Multitarget Stool DNA (mt-sDNA) Use Compared to 1) Patients With BMS Followed by Screening Colonoscopy and No History of mt-sDNA Use and 2) Average-Risk Patients With mt-sDNA Use but No History of BMS

Characteristic	mt-sDNA after BMS	Screening colonoscopy after BMS	Non-BMS mt-sDNA^b^	*P* value mt-sDNA BMS cohort compared to	
	N = 365	N = 139	N = 1542	Screening colonoscopy after BMS	Non-BMS mt-sDNA^b^
Median age, years (IQR)	61 (55–67)	57 (51–64)	67 (61–73)	<.0001	<.0001
Women, n (%)	306 (84)	99 (71)	919 (60)	.002	<.0001
White race, n (%)	357/362 (99)	126/138 (91)	1480/1521 (97)	.0002	.18
Current or former tobacco, n (%)	162 (44)	62 (45)	760/1530 (50)	1.00	.07
History of prior screening colonoscopy, n (%)	209 (57)	NA	906 (59)	-	.64
History of any colorectal neoplasia at prior colonoscopy, n (%)	14/209 (7)	NA	144/862 (17)	-	.0001
Presurgical BMI, kg/m^2^ (IQR)^c^	44 (40–49) (N = 251)	43 (38–48) (N = 138)	-	.14	-
Roux-en-Y gastric bypass, n (%)	297 (81)	102 (74)	-	<.0001	-
Sleeve gastrectomy, n (%)	46 (13)	19 (14)	-		
Vertical band gastrectomy, n (%)	13 (4)	0 (0)	-		
Other BMS^a^, n (%)	9 (2)	18 (13)	-		
History of surgical revision, n (%)	22/361 (6)	6/137 (4)	-	.52	-

IQR, inter-quartile range.

a mt-sDNA pts - Biliopancreatic diversion with duodenal switch (N = 4), Billroth II (N = 1), gastric plication (N = 1), and not otherwise specified (N = 3); Screening pts–Biliopancreatic diversion with duodenal switch (N = 14) and vertical sleeve (N = 4).

b 16 patients with mt-sDNA and prior BMS in this group were removed and analyzed only in the *mt-sDNA after BMS* group.

c BMI data were available for 251 and 138 patients in the *mt-sDNA after BMS* and *screening colonoscopy after BMS* cohorts, respectively.

## Abbreviations used in this paper:

BMI: body mass index
BMS: bariatric and metabolic surgery
CRC: colorectal cancer
EMR: electronic medical record
mt-sDNA: multitarget stool DNA
NSAID: nonsteroidal anti-inflammatory drug
PPV: positive predictive value
