# Supplementary material for: Positive Predictive Value for Multitarget Stool DNA After Bariatric and Metabolic Surgery
Source: Gastro Hep Adv. Author manuscript; Available in PMC 2023 Oct 24. (PMC10597571; doi:10.1016/j.gastha.2023.06.005)
Supplement: 1 [file NIHMS1938346-supplement-1.docx]

Supplemental Table 1: Registry procedure codes for bariatric and metabolic surgery (BMS).

| **Procedure Codes** | **Definition** | **Action after manual chart review** |
| --- | --- | --- |
| 43621 | Gastrectomy | Included and categorized as reflected in Table 1 |
| 43632 | Gastrectomy |  |
| 43633 | Gastrectomy |  |
| 43644 | Gastric Bypass |  |
| 43645 | Gastric Bypass |  |
| 43659 | Bypass NOS |  |
| 43775 | Sleeve gastrectomy |  |
| 43843 | Sleeve gastrectomy |  |
| 43845 | Duodenal switch |  |
| 43846 | Roux-en-Y |  |
| 43847 | Biliopancreatic diversion |  |
| 43848 | Revision NOS |  |
| 43850 | Revision with reconstruction |  |
| 43860 | Revision with reconstruction |  |
| 43770 | **Gastric Band** | Excluded, unless history of invasive bariatric and metabolic surgery |
| 43771 | **Revision of gastric band** |  |
| 43772 | **Removal of gastric band** |  |
| 43773 | **Removal and replacement of gastric band** |  |
| 43774 | **Removal of gastric band** |  |
| 43999 | **Endoscopic bariatrics** |  |
