# Supplementary material for: Positive Predictive Value for Multitarget Stool DNA After Bariatric and Metabolic Surgery
Source: Gastro Hep Adv. Author manuscript; Available in PMC 2023 Oct 24. (PMC10597571; doi:10.1016/j.gastha.2023.06.005)
Supplement: 2 [file NIHMS1938346-supplement-2.docx]

Supplemental Table 2: Neoplastic findings at colonoscopy among patients with bariatric and metabolic surgery (BMS) with multi-target stool DNA (mt-sDNA) utilization versus non-BMS mt-sDNA.

|  | mt-sDNA after BMS  N=47 | non-BMS mt-sDNA  (N=1542) | *P*-value |
| --- | --- | --- | --- |
| **Colorectal Cancer** | 0 (0%) | 14 (1%) | .9999 |
| **Advanced colorectal neoplasia^‡^** | 12 (26%) | 425 (28%) | .8689 |
| Adenoma | 7 (15%) | 258 (17%) |  |
| Sessile Serrated Lesion | 5 (11%) | 167 (11%) |  |
| **≥3 Polyps^ⴕ^ <10mm** | 5 (11%) | 157 (10%) | .8089 |
| Adenoma | 4 (9%) | 117 (8%) |  |
| Sessile Serrated Lesion | 1 (2%) | 40 (3%) |  |
| **1-2 Polyps 5 to 10mm** | 6 (13%) | 162 (11%) | .6274 |
| Adenoma | 3 (6%) | 83 (5%) |  |
| Sessile Serrated Lesion | 3 (6%) | 79 (5%) |  |
| **1-2 Polyps ≤5mm** | 6 (13%) | 278 (18%) | .4417 |
| Adenoma | 4 (9%) | 199 (13%) |  |
| Sessile Serrated Lesion | 2 (4%) | 79 (5%) |  |

‡CRC or adenoma/sessile serrated polyps ≥1cm or with high grade dysplasia or villous elements
