# Supplementary material for: Positive Predictive Value for Multitarget Stool DNA After Bariatric and Metabolic Surgery
Source: Gastro Hep Adv. Author manuscript; Available in PMC 2023 Oct 24. (PMC10597571; doi:10.1016/j.gastha.2023.06.005)
Supplement: 3 [file NIHMS1938346-supplement-3.docx]

Supplemental Table 3: Quality metrics among patients at average risk for colorectal cancer with a history of bariatric and metabolic surgery (BMS) who underwent follow-up colonoscopy versus screening colonoscopy.

|  | **Multi-target stool DNA after Bariatric and Metabolic Surgery (BMS)**  **(N=51**^ǂ^**)** | **Screening Colonoscopy after BMS**  **(N=139)** | ***P*-value** |
| --- | --- | --- | --- |
| **Adequate Bowel Preparation, N (%)** | 45/50 (90%) | 128/139 (92%) | .77 |
| **Cecal intubation, N (%)** | 50 (98%) | 137 (99%) | 1.00 |
| **Withdrawal Time, Minutes (IQR)** | 22 (18-28) (N=16) | 13 (8-19) (N=106) | .0003 |

ǂ multi-target stool DNA after bariatric and metabolic surgery including iron deficiency patients
